# The Analysis of Fincke’s High Potencies by the American Institute

**Published:** 1884-06

**Authors:** B. Fincke

**Affiliations:** Brooklyn, N. Y.


### THE ANALYSIS OF FINCKE'S HIGH POTENCIES BY THE AMERICAN INSTITUTE.

B. FINCKE, M. D., BROOKLYN, N. Y.

*Audiatur et altera pars.*

The Chairman of the Bureau on Microscopy and Histology of the American Institute presented in his report at the session of 1883 three analyses of my high potencies which I propose to criticise as to their correctness and corresponding value.

1. *The samples did not come from me.*—The first analysis is that of "Calc. phos. CM potency from Fincke," and calls for the preliminary question: Where did the analyst obtain that potency? Not from me direct. Then it must have been furnished through somebody else. If the analysis shows any impurities in the sample under examination, as is indeed the case—for the analyst found in this CM potency enough of Silica, Phosphate of Lime, and Iron for a sixth decimal potency—it must be proved that the potency in question came from my hand, and was not dabbled with on the way to the analyst. The following demonstration will raise the suspicion that something must have happened to that potency. If my high potencies are grafted and sold for genuine all over the land, as I can prove, I cannot be made responsible for such practice.

2. *An unaccountable quantity is used.*—My high potencies are all given in the one form of half-drachm vials (so-called, for they are nearly one drachm), containing about forty-two grains of medicated globules, two hundred and five to the grain. But the quantity incinerated by the analyst is ten grams, which amounts to about one hundred and fifty-four grains. Whence came the surplus of one hundred and twelve grains? I prepared Calc. phos. Cm in 1870 and since then four vials in all have gone into the possession of others, here and in England. All these four vials combined would make about one hundred and sixty-eight grains, and here one hundred and fifty-four grains have been used for analysis. Hence this analysis will have to be extended to the source from which the subject of it was obtained.

3. *The methods of testing the residues are not given.*—The analyses of the high potencies do not show the methods employed after incineration, since only the result as to quality and quantity is given. This, of course, precludes any criticism in this direction.

4. *On the Silica and Phosphate of Lime found in the sample.*—After incineration of ten grams a residue of 0.0019 gram is found, consisting of Silica and Phosphate of Lime enough for the sixth decimal, and of Iron 0.00004 gram, enough for the sixth decimal. From what source this Silica and Phosphate of Lime has come is not for me to explain, because I am innocent of it. I know that when I started the fluxion with a drop of the sixth initial potency of Calc. phos., which in the opinion of the analyst would represent the billionth grain centesimally, there could not have been any larger amount of Calc. phos. in it than the one billionth of a grain. As to the Silica, I am at a loss to see where that could have come from. There is nothing to account for it, neither in the preparation of the first six initial potencies made by hand, nor in the fluxion process following. The impurities, therefore, could have no similar source as those shown in the analyst's previously given analysis of sugar of milk, triturations, and pellets.*

Where the Iron came from is explainable by the fact that it appears in all three analyses, and also in those previously given, with nearly the same amount, which points to a common impurity of the pellets, but not to an impurity of the potencies. Finally, the possibility that the original crude substance from which the potency was made might have contained these impurities in an infinitesimal amount can hardly be adduced as a proof that this analysis of the CM is correct which finds in it enough for the sixth decimal—even two and three times enough. The justness of the rejection of this analysis must be still more evident to the

* Some of my pellets of Calc. phos. CM have been tested spectroscopically and no line was found in the spectrum except the faint sodium line from the atmosphere.

analyst himself, when it is remembered how the fluxion method has been assailed by his friends for the reason that every particle of medicine contained at first in the potentiating vial is washed out entirely, so that from a certain period nothing remains but water to the end. If there is any value in this view, it is that it is contradictory to the analysis of "Calc. phos. CM from Fincke."

5. *The analyses prove the purity of my high potencies.*—The following two analyses of Arsen. a. M and Merc. viv. Cm confirm the valuelessness of the same for showing impurities in the high potencies. They show only traces of Iron, enough two and three times for the sixth decimal. Traces of Iron to a similar amount have also been discovered in the pellets from other sources which had not been medicated. There is then, as it seems, the fault with the pellets. And as no other substance was found in my high potencies, the inference is that they were pure and contained nothing but the potency, and thus these analyses prove the contrary of what they intend to show.

6. *The manufacturers' judgment of the analysis of the pellets.*—Now, these globules have been manufactured by the old and celebrated Clarkson Homœopathic Globule Company, in Troy. Their fabric has always been the most pure and satisfactory for the purpose of administering homœopathic remedies.

"Our globules," they write to me, "are made of pure cane sugar and nothing else. We use the very best and whitest we can obtain, and manufacture nothing else. We have a light and very clean manufactory on the river-bank and use the greatest pains to give the doctors a good and reliable pellet. As far as we know no iron is used in the manufacture unless it is the poker he rakes his fire with, or the tin roof. We should think a doctor carrying his investigations so far would meet obstacles at every turn. The water that is put in gruel or beef tea must have an effect much greater than the impurities in the sugar. We know how important it is to have medicine and the globules free from impurities, and we use our utmost endeavors to have them so. The vessels we use are of copper, and we think there is nothing about them that could have an effect."

This is an unsophisticated judgment of the analysis in question.

7. *The chemical tests of the pellets show a very faint trace of Iron.*—A very faint trace of Iron, however, is found in the pellets from Clarkson (which I commonly use) by the following chemical tests of Mr. C. George Seiboth, assistant of Mr. A. Grund, analytical chemist, 121 Front Street, New York.

The test tubes were first cleaned and thoroughly washed with distilled water, and on testing them chemically they were found to be perfectly clean.

*First Test.*—About fifty grains of pellets were dissolved in sufficient distilled water to produce a concentrated solution. This solution was then attenuated by adding more distilled water in a test tube. A few drops of chemically pure Nitric acid were added and the mixture was boiled over a Bunsen burner. On adding to it the Sulphocyanide of Potassium, a very pale pink color was observed, proving the presence of Iron.

*Second Test.*—The concentrated solution of sugar pellets treated by attenuation with distilled water as before, gave on addition of the yellow Prussiate of Potash a faint trace of a very pale greenish-blue color, confirming the former test.

The trace of Iron claimed for my high potencies by the analyst of the Institute, then, comes from the sugar of the pellets which have been medicated with them.

8. *This trace of Iron is not an antidote to the potencies.*—The inferences drawn from the presence of such a minutular trace of crude Iron in the pellets must be rejected as unjustifiable and false. For, if such pellets have been used as vehicles for the potency which is to be administered to the patients, they can in no way affect the potency. The potency has been carried up to the high degree desired *lege artis*, and preserved in alcohol. Of this alcoholic tincture a few drops are added to the globules in a half-drachm vial, which by capillary attraction soak the fluid into their substance. To make out that the amount of 0.000-002 gram of crude Iron act as antidote to the millionth centesimal potency of Arsen. a. is as preposterous as to say that the crude Iron contained in our drinking-water to a much greater amount, in which we dissolve a few pellets of this remedy or of Merc. viv. Cm will neutralize their effect, because the Sesquioxide of Iron is an antidote to Arsenic poisoning, and Iron is said to be an antidote to Merc. viv., which is to be proved yet.

9. *A fraction of crude substance is not a homœopathic potency.*—It appears that the analyst has as yet a crude idea of potentiation when he maintains that such a minute fraction of crude matter as 0.000002 gram be equal to twice enough for the sixth des. or third cent. He seems to think that a millionth part of a gram is the same third cent. potency, as if a grain of that substance is attenuated by means of an inert vehicle in the proportion of one to hundred three times under the manual assistance of trituration and succussions according to Hahnemann's rule. The analyst to see his error would have to try a simple one-millionth grain of crude Iron of his own preparation without vehicle upon a suitable subject and see what it will accomplish, and then, as a counterpart, test a third centesimal potency prepared according to Hahnemann, and he would mark a difference. The difference would be still greater in the higher potencies and he would find that it is as inadmissible to call a millionth grain of crude Iron the sixth dec. as a decillionth of it the thirtieth centesimal potency.

10. *A seven-hundred-millionth of a grain of crude Iron is insignificant in a pellet used as vehicle for high potencies.*—Besides, this manner of calculating the impurity contained in the vehicle as a fifth or sixth decimal potency is not correct. If there is sense in it, it is, that these potencies are extant only "potentia," not "actu." The analyst figures them out in his brain, but they are not in the potency which he is analyzing in reality; they are only a figment of his brain and have only psychological reality. The use made of it, in order to make the higher potencies appear lower than they are, is not justified from a scientific standpoint.

Assuming that the pellets contain in one grain the largest quantity given in the analysis of my high potencies, 0.00004 gram, how much will that be upon one pellet given to the patient for a dose? One vial contains 42 grains of pellets, at 205 a grain; hence the vial contains 8,610 pellets. 0.00004 gram=0.0006168 grain, divided by 8,610 pellets, give, therefore, for one single pellet, the formidable weight of 0.00000007 grain of crude Iron. Is it worth while to cast a shadow upon high potencies by pretending that the one pellet contains an impurity of seven-hundred-millionths parts of a grain of crude Iron—enough, in the opinion of the analyst, for a fourth centesimal potency, antidoting the medicine for which it is used as vehicle—when we take much greater quantities daily in our food and drinking-water? And what quantities do the analyst and his friends use in their daily practice?

If the analyst is correct, he must prove that when a Cm is given, together with the drinking-water, this seven-hundred-millionth grain of crude Iron will antidote the potency (M), and hence contaminate its curative and probative effects.

The difference in magnitude (or minuteness) and condition between a seven-hundred-millionth grain of crude Iron in a dose of one pellet and a Cm, expressed by a decimal fraction with two hundred ciphers and a M with a decimal fraction with two million ciphers, is so great that the assumed significance is quite insignificant.—*Matth.* 23, 24.

11. *Inadequate application of the analysis of crude sugar of milk and triturations to my high potencies.*—It need hardly be pointed out that "the analysis of Calc. phos., sixth decimal triturations," and "the impossibility of accomplishing the higher triturations of Phosphate of Lime, using sugar of milk as the triturating vehicle," has been made use of only to throw suspicion on the preparation of a Cm of the same remedy by Fincke.

They have nothing whatever to do with it and are put in this place—where they do not necessarily belong—merely for effect.

12. *Cane-sugar is not a vehicle in potentiation by fluxion.*—The application of the analysis of sugar of milk upon the use of cane-sugar as a vehicle in the higher potencies, ingenious as it may be, is likewise by no means justifiable. For the analyst, as a homœopathic physician, must know that cane-sugar is not used as a vehicle in preparing the higher potencies, such as sugar of milk, water, and alcohol is used in the process of potentiation. In fairness he ought to have said: "In the higher and lower potencies;" for the use as a vehicle in the higher potencies is in no way different from its use in the lower potencies. Nay—in the lower potencies its application in large quantities is much more marked; not only globules are used, but globes, lozenges, disks, tablets, and what not—such as we do not use in the practice with high potencies, where we avail ourselves of the beautiful Hahnemannian invention of the smallest globules, of which only a minute quantity of one or more are given for a dose.

13. *The investigation of sugar of milk triturations, cane-sugar, and pellets is to be encouraged.*—In the foregoing I have only examined the remarks of the Chairman on microscopy as far as he has analyzed my high potencies. The rest of his investigations concerns the crude sugar of milk and the preparations of the lower potencies in the form of decimal triturations with sugar of milk up to the thirtieth, such as are bought in the shops. It is very laudable that this time the names are given, so that everybody knows where to get the best sugar of milk and triturations made with it.

By all means let this investigation go on. This is the legitimate task for those who adhere so far to the freedom of medical opinion and action as to apply in their practice crude substances and low potencies, decimal dilutions and triturations. It will probably ultimately lead to the preference of high potencies which are not loaded down with the manifold impurities which the diligent analyst of the Institute has encountered. They are products of the natural evolution of the Hahnemannian ideas, which are founded, not upon the physical sciences, but upon the laws of life.

*Ceterum censeo, macrodosiam esse delendam.*

